# A model of crosslink kinetics in the expanding plant cell wall: yield stress and enzyme action

**DOI:** 10.1016/j.jtbi.2012.04.035

**Published:** 2012-08-21

**Authors:** R.J. Dyson, L.R. Band, O.E. Jensen

**Affiliations:** a School of Mathematics, The Watson Building, University of Birmingham, Edgbaston, Birmingham B15 2TT, UK; b Centre for Plant Integrative Biology, University of Nottingham, Sutton Bonington, LE12 5RD, UK; c School of Mathematical Sciences, University of Nottingham, University Park, Nottingham NG7 2RD, UK

**Keywords:** Cellulose microfibril, Hemicellulose, crosslink, yield stress

## Abstract

The plant primary cell wall is a composite material containing stiff cellulose microfibrils that are embedded within a pectin matrix and crosslinked through a network of hemicellulose polymers. This microstructure endows the wall with nonlinear anisotropic mechanical properties and allows enzymatic regulation of expansive cell growth. We present a mathematical model of hemicellulose crosslink dynamics in an expanding cell wall incorporating strain-enhanced breakage and enzyme-mediated crosslink kinetics. The model predicts the characteristic yielding behaviour in the relationship between stress and strain-rate seen experimentally, and suggests how the effective yield and extensibility of the wall depend on microstructural parameters and on the action of enzymes of the XTH and expansin families. The model suggests that the yielding behaviour encapsulated in the classical Lockhart equation can be explained by the strongly nonlinear dependence of crosslink breakage rate on crosslink elongation. The model also demonstrates how enzymes that target crosslink binding can be effective in softening the wall in its pre-yield state, whereas its post-yield extensibility is determined primarily by the pectin matrix.

## Highlights

► We present a model of crosslink dynamics in an expanding plant cell wall ► Yield can be explained by the dependence of crosslink breakage rate on elongation. ► Enzymes that target crosslink binding can soften the wall in its pre-yield state.

## 1 Introduction

Plant cells are surrounded by a tough primary cell wall which maintains a high internal turgor pressure while allowing significant anisotropic expansion. Such growth is driven by irreversible stretching of the cell wall under the action of the turgor pressure, with the growth rate being dependent on the mechanical properties of the cell wall. The plant primary cell wall is a composite material containing stiff cellulose microfibrils (CMF), embedded within a pectin matrix and linked through a network of hemicellulose crosslinks (Carpita and Gibeaut, 1993; Cosgrove, 2005). This structure exhibits mechanical anisotropy because the CMF are typically orientated in a preferred direction, making the wall much less extensible in a direction parallel to the CMF than perpendicular to them (Baskin, 2005; Suslov and Verbelen, 2006; Van Sandt et al., 2007). Stresses acting perpendicular to the CMF (and in the plane of the wall) are shared between the hemicellulose network and the pectin matrix, with some authors suggesting the former are dominant in some circumstances (Van Sandt et al., 2007; Vissenberg et al., 2000). During growth, it is thought that new wall material is continually deposited on the inner face of the wall to maintain its integrity (Cosgrove, 2005; Vissenberg et al., 2000). To understand plant cell growth and its regulation, we must therefore determine how the properties of this evolving composite structure relate to the macroscale mechanical properties of the cell wall (see Burgert and Fratzl, 2007; Cosgrove, 2000; Geitmann, 2010; Geitmann and Ortega, 2009 for recent reviews).

Many researchers have followed Lockhart (1965) in modelling the cell wall at the macroscopic level as an anisotropic Bingham material, displaying a yield stress *Y* below which no irreversible deformation occurs and an extensibility Φ which determines how the cell (or tissue) elongation rate relates to the driving stress, provided this stress exceeds *Y*. To illustrate, consider an isolated circular cylindrical cell of length ℓ(t^*^) and radius *R* at time t^*^ having CMF oriented in a hoop-like manner orthogonal to the axis of the cell, preventing radial expansion. The turgor pressure *P*, acting on the flat end-plates of the cell, generates a force *πR*^2^*P* that is balanced by the axial stress resultant (or tension) Σ^*^ in the curved cell wall (distributed around the cell perimeter 2*πR*), so that $\Sigma^{*}=\frac{1}{2}\mathrm{RP}$; the hoop stress resultant *RP* is borne by the CMF. The Lockhart equation (Lockhart, 1965) describing the expansion of the cell may then be written as

(1) $$\frac{1}{\ell }\frac{d\ell }{dt^{*}}=\left\{\begin{array}{cc} 0 & (\Sigma^{*}<Y),\\ \Phi (\Sigma^{*}-Y) & (\Sigma^{*}>Y). \end{array}\right)$$

Experimental studies have found the Lockhart equation (1) to be a reasonably good description of plant cell-wall mechanics when water fluxes needed to maintain cell turgor are not rate-limiting (see for example, Green et al., 1971). Variants of (1) can be used to describe the elongation of sections of cell wall, whole cells or multicellular tissues, making it a natural building block in integrative multiscale models of plant growth and development (Chavarrí a-Krauser et al., 2005; Chickarmane et al., 2010; Mirabet et al., 2011). Note that instead of using Σ^*^, (1) may be expressed in terms of turgor pressure *P* or the extensional stress Σ^*^/*h* (where *h* is the cell wall thickness), with the definition of extensibility and yield being adapted accordingly by incorporating appropriate geometrical factors, indicating the importance of cell and tissue geometry in determining plant growth rate. The Lockhart equation (1) can be interpreted in two equivalent ways, making it a particularly powerful tool in describing plant growth: it may be read from left to right as growth rate being determined by the internal stresses within the plant tissue, modulated by cell and tissue properties; equivalently it can be read from right to left as a statement of the constitutive properties of cell wall or plant tissue, describing how material stress is related to strain rate.

A number of previous studies have addressed the relationship between the empirical parameters Φ and *Y* and the cell wall's microstructure, and the broader applicability of (1). For example, Ortega and co-workers showed how strain-hardening may arise via recruitment of hemicellulose crosslinks (as reviewed in Geitmann and Ortega, 2009). Dyson and Jensen (2010) derived a version of (1) from a continuum mechanics model of the wall of the elongating cell, treating the wall as a thin sheet of viscous fibre-reinforced fluid. Their model demonstrates explicitly how, when the CMF are orientated perpendicular to the axis of the cell, the extensibility is determined by a viscosity that characterises the pectin matrix (and the embedded CMF and hemicellulose crosslinks). Similar conclusions about the importance of the properties of the pectin-hemicellulose matrix have been reached in (Carpita and Gibeaut, 1993; Dumais et al., 2006). When the CMF are not oriented perpendicular to the cell axis, the fibres may reorient passively as the cell elongates (following the so-called ‘multi-net model’ (Preston, 1982)), in which case more complex expressions of the form

(2) $$\Sigma^{*}=f(\alpha^{*},\ell ),\;\alpha^{*}\equiv \frac{1}{\ell }\frac{d\ell }{dt^{*}}$$

can arise; *f* is a nonlinear function of the cell length ℓ and the elongation rate α^*^. (Here we adopt the convention common in the continuum mechanics literature and write stress as a function of strain or strain-rate.) Such a function was derived in (Dyson and Jensen, 2010) to show how growth may be suppressed by fibre reorientation, and alternative functions may be used to mimic viscoelastic behaviour (Geitmann and Ortega, 2009), as is necessary to model the rapid response of a cell to a sudden change in its external loading. Note that in the case of the classical Lockhart equation (1), *f* is independent of ℓ and is linear in α^*^ when α^*^ > 0, such that the ‘effective extensibility’ $\Phi_{\mathrm{eff}}^{*}$, defined here by

(3) $$\Phi_{\mathrm{eff}}^{*}\equiv {\left(\frac{d\Sigma^{*}}{d\alpha^{*}}\right)}^{-1},$$

takes the values $\Phi_{\mathrm{eff}}^{*}=0$ in the pre-yield state (Σ^*^ < *Y*) or $\Phi_{\mathrm{eff}}^{*}=\Phi$ in the post-yield state (Σ^*^ > *Y*).

A few previous models address the mechanical properties of the interacting cellulose-hemicellulose network (Kha et al., 2010; Passioura and Fry, 1992; Veytsman and Cosgrove, 1998). Passioura and Fry (1992) consider a simple model in which it is assumed that crosslinks extend as they gradually detach from the CMF, become progressively load-bearing as the wall stretches (assuming the number of load-bearing tethers is proportional to the distance between CMF) and rupture according to a time-dependent law. Their model assumes uniform properties across the cell wall, while acknowledging Preston's observation (Preston, 1982) that CMF are carried towards the outer surface of the elongating cell wall. They assume each bond behaves like a Bingham element (with a yield stress), and propose a relation between the macroscopic yield and the molecular yield parameter. The computational WallGen model (Kha et al., 2010) predicts anisotropic elastic properties from a virtual cell wall assembled from individual polymers, but this model does not allow for crosslink breakage, growth or irreversible viscous deformation of the cell wall. Veytsman and Cosgrove (1998), developing the concept of the ‘sticky network’ model (Cosgrove, 2000), use a thermodynamic formulation to relate the elastic stress in the composite cell wall to the properties of the CMF and hydrogen bonds between the CMF and glucan (hemicellulose) molecules, predicting the existence of an elastic yield stress above which the cell wall will exhibit creep. However their model does not capture the anisotropic stresses arising in ordered polymer networks (for which CMF have a predominant orientation, which may be orthogonal to the direction in which the wall elongates), nor the deposition of new material into the cell wall nor viscous stresses associated with crosslink detachment and reformation.

**Fig. 1:**
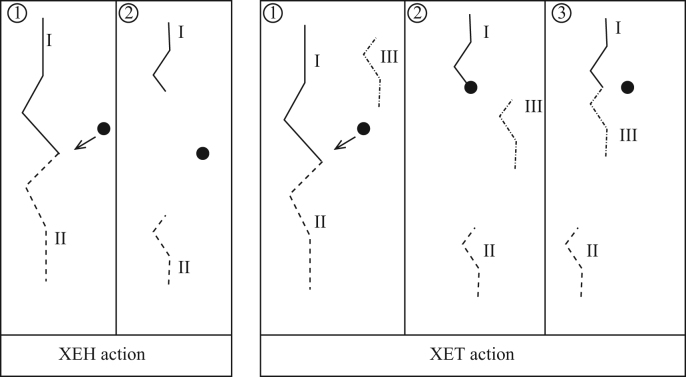
Two modes of enzyme action on the hemicellulose crosslinked network. Under XEH action (with enzyme denoted by a black circle), the hemicellulose crosslink is broken leaving two free ends. Under XET action the hemicellulose crosslink is broken, and one of the resulting free ends is joined to a third free end within the wall.

These previous descriptions of cell-wall mechanics have typically not considered the role of enzymes. During growth, the cell wall's structure is thought to be modified by various remodelling enzymes, different families of which act on different components of the cell wall. Pectin methyl esterase (PME) affects the consistency of the pectin ground matrix, removing methyl groups by breaking ester bonds. This enables pectin to be crosslinked by calcium ions, stiffening the cell wall and reducing cell expansion (Boyer, 2009; Derbyshire et al., 2007; Proseus and Boyer, 2006); recent progress has been made in quantifying PME action in a chemorheological model of the pectin matrix (Rojas et al., 2011). Some members of the XTH enzyme family loosen the wall via XEH (xyloglucan endohydrolase) activity, which involves breaking the bond between two hemicellulose crosslinks, whereas other members of this family carry out XET (xyloglucan endotransglucosylase) action, whereby the crosslink is broken and then one free end is rejoined to another free crosslink end within the tissue (see Figure 1) (Rose et al., 2002). In addition, expansins break the hydrogen bonds between the CMF and the hemicellulose strands (McQueen-Mason et al., 1992). The different remodelling enzymes are therefore likely to affect the macroscale cell-wall properties, and hence the cell's growth rate, in different ways. Hormonal regulation of plant growth is thought to be in part via regulation of these remodelling enzymes (Catala et al., 1997; Potter and Fry, 1993; Redgwell and Fry, 1993; Smith et al., 1996; Wu et al., 1994; Xu et al., 1996, 1995; Zurek and Clouse, 1994), so that determining how the enzymes affect the cell wall's mechanical properies is vital to gain understanding of the interplay between growth regulation at different spatial scales. As far as we are aware, no previous model has considered the role of XTH and only the model developed in (Pietruszka, 2011) has included expansin effects, via a modification of the Lockhart equation through a time-dependent extensibility.

The relative importance of the different wall components and remodelling enzymes will vary between plant tissues and remains a matter of debate. However we focus here on the hemicellulose crosslinks and the action of XTH and expansin. Building on previous micromechanical studies (Dyson and Jensen, 2010; Passioura and Fry, 1992), and exploiting some simple ideas from transient network theory for viscoelastic polymer solutions (Green and Tobolsky, 1946; Larson, 1988; Yamamoto, 1956) and gels (Groot et al., 1996) (a theory which has previously been successfully used to describe fibrous biological materials such as ligaments and tendons (Ciarletta and Amar, 2009)), we seek here to relate crosslink kinetics, mediated by enzyme action, to the macroscopic mechanical properties of a section of cell wall, which can then be scaled up to describe the elongation of an individual cell or a whole tissue. Our model enables us to evaluate the applicability of the Lockhart equation (1) and suggests how to parameterize the effects of XTH and expansin enzyme action in multiscale models of plant growth, which in some circumstances may require the full machinery of computational mechanics (Huang et al., 2012). In particular, our model suggests that the kinetics of strain-enhanced crosslink breakage plays a significant role in determining the cell wall's yield stress *Y* and that enzymes that target crosslinking may soften the wall in its pre-yield state. In contrast to previous studies, our model incorporates length-dependent populations of the hemicelluose crosslinks, it resolves differences in crosslink populations across the cell wall and it distinguishes XEH, XET and expansin modes of enzyme action.

## 2 A model for the expanding cell wall

In Section 2.1 & 2.2 we describe a model for the transient response of a section of cell wall under an imposed force in the absence of enzyme action, in order to relate crosslink kinetics to stress relaxation and yield effects in the wall. We then incorporate XTH enzyme action (Section 2.3), restricting attention to steady-state configurations. Our model for expansin action is described in B. The modelling framework is deliberately simple, aiming to provide qualitative mechanistic explanations for observed behaviour in terms of the evolving microstructure. By addressing a simple geometry we avoid complex tensor descriptions of cell wall material. Predictions of the model are reported in Section 3 below.

### 2.1 Model derivation: transient evolution

**Fig. 2:**
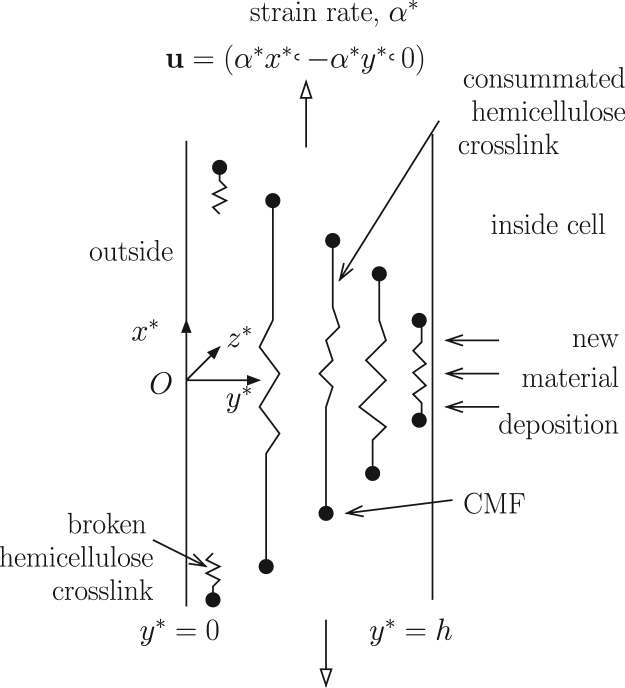
A cross-section of the cell wall, where the CMF are represented as black circles and lie perpendicular to the page, showing the movement of hemicellulose crosslinks through the cell wall.

We consider the evolution of a segment of cell wall that elongates via uniform stretching (Figure 2). The cell wall contains CMF that run orthogonal to the cell axis and to the direction of cell elongation; crosslinks connecting adjacent CMF provide resistance to elongation. The wall is initially at rest for time t^*^ < 0; in t^*^ > 0 it undergoes steady stretching at a constant rate α^*^, induced by some externally imposed force. This formulation allows us to investigate both short-term elastic effects and long-term viscous effects. We wish to determine the evolution of the wall microstructure, and particularly the development of stress in the wall as a result of crosslink extension, breakage and rebinding. The wall strain rate α^*^ can be related to the length of the cell in which the wall segment is embedded via (2)_2_. We assume that the wall thickness *h* is substantially smaller than the cell radius, allowing the curvature of the cell wall to be neglected. We follow (Dyson and Jensen, 2010) in decoupling hoop stresses acting along CMF, which balance turgor pressure acting normally on a curved wall, from the axial stress resultant Σ^*^(t^*^) that drives elongation of the cell wall. We wish to relate Σ^*^(t^*^) to α^*^.

We introduce a coordinate system (x^*^, y^*^, z^*^) with its origin *O* fixed to a material point on the outer surface of the cell wall, such that the wall occupies 0 ≤ y^*^ ≤ *h*, the wall stretches in the x^*^-direction with strain rate α^*^ and the CMF are oriented in the z^*^-direction (Figure 2). Adjacent CMF are connected by crosslinks, each of which is assumed to lie (at least predominantly) in a plane z^*^ = constant. We assume the CMF are embedded strongly in the matrix, i.e. we assume affine deformations of the CMF and crosslinks. In the neighbourhood (and frame of reference) of *O*, the motion of the wall for t^*^ > 0 can be represented via the incompressible velocity field

(4) $$\text{u}=\alpha^{*}\left(x^{*},-y^{*},0\right),\;\left(0<y^{*}<h\right),$$

so that stretching of wall material in the x^*^-direction balances compression in the y^*^-direction. (This velocity field is typical of extensional flow of a viscous sheet (Howell, 1996): if one assumes the axial velocity component u^*^ relative to *O* is uniform through the sheet with elongation rate *α*, so that ∂u^*^/∂x^*^ = α^*^, then incompressibility requires that $\partial v^{*}/\partial y^{*}=-\alpha^{*}$, from which (4) follows.) The wall does not stretch in the fibre (z^*^-) direction and henceforth we need consider only two-dimensional motion in the (x^*^, y^*^)-plane. We assume that, for t^*^ > 0, new wall material (a mixture of CMF, hemicellulose and pectin) is continually deposited on the inner surface of the cell wall at y^*^ = *h* at a rate that maintains a constant cell-wall thickness.

As the wall elongates, newly deposited material is carried through the wall towards its outer surface via (4). The density of any passive non-diffusing cell-wall component, *ρ*(x^*^, y^*^, t^*^), evolves according to

(5) $$\frac{\partial \rho }{\partial t^{*}}+\text{u}\cdot \nabla \rho =0\;\mathrm{with}\;\rho (x^{*},h,t^{*})=\rho_{0};$$

*ρ*_0_ is the density at which material is deposited. Thus d*ρ*/dt^*^ = 0 on the characteristics dx^*^/dt^*^ = α^*^x^*^, dy^*^/dt^*^ = − α^*^y^*^, so that material initially located at (x^*^, y^*^) = (*x*_0_, *h*) at t^*^ = *τ* (for some *x*_0_ and *τ* ≥ 0) is convected along $x^{*}=x_{0}e^{\alpha^{*}(t^{*}-\tau )}$, $y^{*}={\mathrm{he}}^{-\alpha^{*}(t^{*}-\tau )}$ for t^*^ > *τ*, i.e. *ρ* = *ρ*_0_ along the streamline x^*^y^*^ = *x*_0_*h*. It follows that if *ρ*(x^*^, y^*^, 0) = *ρ*_0_ in 0 < y^*^ < *h*, then the density remains uniform throughout the wall thereafter. We can therefore assume that the densities of CMF, hemicellulose and pectin matrix are homogeneous throughout the wall as it stretches, provided the density of deposition of each material matches its initial density.

Under the flow (4), the CMF remain oriented in the z^*^-direction and crosslinks lying in the plane z^*^ = 0 (say) are stretched in the x^*^ direction but compressed in the y^*^-direction. Crosslinks oriented in the y^*^-direction are therefore mechanically insignificant as t^*^ increases and we need consider only those oriented in the x^*^-direction. Suppose such a crosslink has length *L*_*i*_ at time *t*_0_ ≥ 0, when it lies along $y^{*}=y_{i}^{*}$ (for some $0<y_{i}^{*}\leq h$). Under (4), and provided it does not break, the crosslink length L^*^(t^*^) evolves according to

(6) $$\frac{dL^{*}}{dt^{*}}=\alpha^{*}L^{*}\;\mathrm{on}\;\frac{dy^{*}}{dt^{*}}=-\alpha^{*}y^{*}.$$

Thus $L^{*}=L_{i}e^{\alpha^{*}(t^{*}-t_{0})}$ on $y^{*}=y_{i}^{*}e^{-\alpha^{*}(t^{*}-t_{0})}$, i.e. $L^{*}=L_{i}y_{i}^{*}/y^{*}$; the crosslink elongates as it approaches the outer surface of the wall (y^*^ → 0). Let *L*_0_ be the length of crosslinks when unstressed. We assume for simplicity that at t^*^ = 0 all crosslinks have length *L*_0_, as do all newly deposited crosslinks on y^*^ = *h* for t^*^ > 0. Crosslinks are then immediately load-bearing as they start to stretch with the wall (in contrast to the model in Passioura and Fry, 1992).

Stretching will promote crosslink breakage, so the number density of consummated crosslinks (per unit area of cell wall), n^*^(y^*^, t^*^), will vary with y^*^ but may be assumed uniform in x^*^, being given by a Smoluchowski equation (Yamamoto, 1956) of the form (neglecting Brownian fluctuations)

(7) $$\frac{\partial n^{*}}{\partial t^{*}}-\alpha^{*}y^{*}\frac{\partial n^{*}}{\partial y^{*}}=-k_{\mathrm{off}}^{*}\;n^{*}.$$

In the absence of enzymes, we assume the breakage rate is

(8) $$k_{\mathrm{off}}^{*}=k_{0}\exp\left(\beta^{2}\frac{\kappa {\left(L^{*}-L_{0}\right)}^{2}}{2k_{b}T}\right),$$

where *k*_*b*_*T* is the unit of thermal energy (*k*_*B*_ being Boltzmann's constant and *T* absolute temperature), *k*_0_ is the breakage rate of unstressed crosslinks and the breakage rate under force is enhanced according to the model of Dembo et al. (1988) (although other models may be considered, and indeed have been explored in other contexts (Fuller and Leal, 1981; Guy, 2004)). The energy in a stretched crosslink, $\frac{1}{2}\kappa {\left(L^{*}-L_{0}\right)}^{2}$, normalised by *k*_*b*_*T*, is modelled by treating each crosslink as a linear spring with stiffness *κ*. *β* is an empirical parameter that characterises the curvature of the potential well in the crosslink's energy landscape (discussed further below). We also assume

(9) $$n^{*}(h,t^{*})=n_{0},\;n^{*}(y^{*},0)=n_{0}\;(0<y^{*}\leq h),$$

implying that new crosslinks are deposited at the inner face of the wall at constant number density *n*_0_, equal to the density prior to stretching. In practice, crosslink formation can be expected to occur throughout the wall, which would then demand the use of an age-structured model. We sidestep this additional complexity (and the associated closure problem) by decoupling crosslink breakage and formation, assuming all crosslinks form at the inner surface of the wall via (9).

The total stress resultant in the wall (in the direction of elongation) has two components acting in parallel: that generated by the hemicellulose crosslinks within the cell wall and that due to the stretching pectin matrix, i.e.

(10) $$\Sigma^{*}\left(t^{*}\right)=\int_{0}^{h}n^{*}\left(y^{*},t^{*}\right)\kappa \left(L^{*}(y^{*},t)-L_{0}\right)\;dy^{*}+\frac{\alpha^{*}}{\Phi_{m}},$$

where Φ_*m*_ is a constant representing the pectin matrix extensibility. Φ_*m*_ may be estimated in terms of the Trouton extensional viscosity of a sheet of thickness *h* as 1/Φ_*m*_ = 4*μh*, where *μ* is the shear viscosity of the matrix material (modified by the CMF and hemicellulose volume fractions) (Dyson and Jensen, 2010); we ignore any yield stress of the matrix in the present study. We wish to establish how the baseline extensibility Φ_*m*_ is modified by crosslink kinetics, represented by the integral in (10).

To summarise, equations (6)–(9) describe the number density of crosslinks as a function of the distance through the cell wall. Equation (10) provides the corresponding total stress resultant and hence characterises the macroscale mechanical behaviour of the cell wall. We seek the stress resultant Σ^*^ in terms of the strain rate and material parameters.

### 2.2 Model analysis: transient evolution

It is convenient to nondimensionalise the system using

(11) $$\begin{array}{c} n^{*}=n_{0}n,\;y^{*}=hy,\;y_{i}^{*}=hy_{i},\;L^{*}=L_{0}L,\\ t^{*}=t/k_{0},\;\alpha^{*}=k_{0}\alpha ,\;\Sigma^{*}=E\Sigma ,\;k_{\mathrm{off}}^{*}=k_{0}k_{\mathrm{off}} \end{array}$$

and defining

(12) $$E\equiv n_{0}\kappa L_{0}h,\;\sigma \equiv \frac{\kappa L_{0}^{2}}{2k_{b}T},\;\Gamma \equiv \frac{k_{0}}{\Phi_{m}E}.$$

E is a (dimensional) measure of the extensional stiffness of the wall due to crosslinks while *σ* and Γ are dimensionless parameters. We can then re-express (6)–(9) in dimensionless form as

(13a) $$\frac{dL}{dt}=\alpha L,\;\frac{dn}{dt}=-n\exp\left(\beta^{2}\sigma {\left(L-1\right)}^{2}\right),$$

on d*y*/d*t* = − *αy*, with

(13b) L(1,t)=n(1,t)=1,L(y,0)=n(y,0)=1.

The dimensionless stress resultant,

(13c) $$\Sigma \left(t\right)=\int_{0}^{1}n\left(y,t\right)\left(L-1\right)\;dy+\Gamma \alpha ,$$

depends on the dimensionless parameters *β*, which regulates the enhancement of crosslink breakage rate under applied strain, Γ, the relative extensibility of crosslinks to matrix and *α*, the strain rate scaled on the crosslink breakage rate.

If each crosslink molecule is modelled as a Gaussian chain of *N*_*k*_ links each of length *b*, for which stiffness arises from a change in entropy on elongation, then $\sigma =3L_{0}^{2}/2{\langle L\rangle }_{0}^{2}$ where the average squared end-to-end distance in the unstressed configuration is ${\langle L\rangle }_{0}^{2}=N_{k}b^{2}$ (Larson, 1988). *σ* is therefore likely to be of order unity and we can therefore set *σ* = 1 without loss of generality. We estimate *β* by comparing the average CMF spacing (10-20 nm Jarvis, 2009), which provides an estimate of *L*_0_, with the length *L*_max_ of fully stretched crosslinks (reported as up to 400nm Burgert and Fratzl, 2007; McCann et al., 1990, 1992). Thus to allow crosslinks to extend significantly before they break, we widen the potential well in (8) by taking *β* ≪ 1. More precisely, we can identify the ratio *L*_max_/*L*_0_ = *O*(1/*β*), i.e. the breakage rate in (13a) rises steeply when *L* = *O*(1/*β*).

**Fig. 3:**
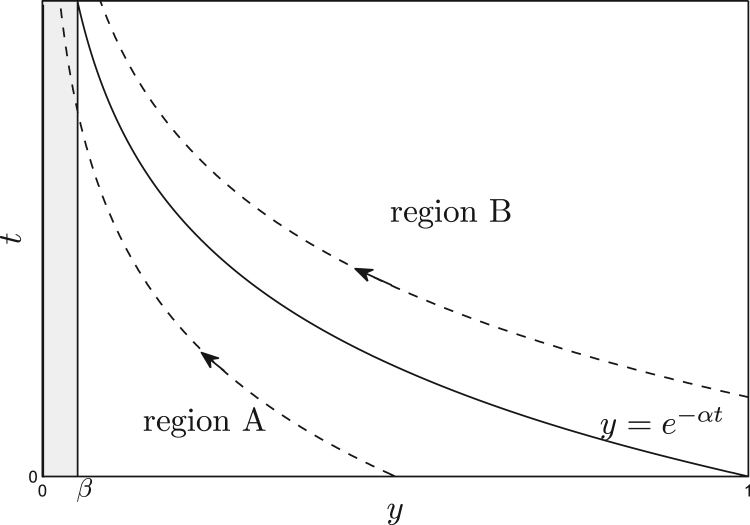
A schematic characteristics diagram displaying the two different regimes: the evolution of the initial wall configuration (region A) and the the evolution of crosslinks deposited on *y* = 1 during the motion (region B). The shaded region illustrates the scission layer arising when *β* ≪ 1.

The solution of (2.2) involves two groups of crosslinks: those present at *t* = 0 in 0 ≤ *y* ≤ 1; and those deposited on *y* = 1 in *t* > 0 (see regions A and B in figure 3). Each crosslink in the former group lies on a characteristic *y* = *y*_*i*_*e*^−*αt*^, parameterized by its initial position 0 < *y*_*i*_ ≤ 1, satisfying (from (13a))

(14) $$\frac{dn}{dL}=-\frac{n}{\alpha L}\exp\left(\beta^{2}{\left(L-1\right)}^{2}\right).$$

For this group, in 0 < *y* < *e*^−*αt*^, it follows (using (2.2b)_2_) that

(15) $$n=\exp\left(-\frac{G(1/L)}{\alpha }\right),\;L=e^{\alpha t},$$

where

(16) $$G(y)\equiv \int_{y}^{1}\frac{\exp\left(\beta^{2}{\left[(1/z)-1\right]}^{2}\right)}{z}\;dz.$$

The other group of crosslinks, deposited on *y* = 1 for *t* > 0, satisfy (14) and (2.2b)_1_ so that

(17) $$n=\exp\left(-\frac{G\left(1/L\right)}{\alpha }\right),\;L=1/y$$

in the expanding domain *e*^−*αt*^ ≤ *y* ≤ 1. Partitioning the integral in (13c) over regions A and B, the stress resultant is therefore given by

(18) $$\Sigma \left(t\right)=\exp\left(-\frac{G(e^{-\alpha t})}{\alpha }\right)\left(1-e^{-\alpha t}\right)+\int_{e^{-\alpha t}}^{1}\exp\left(-\frac{G(y)}{\alpha }\right)\left(\frac{1}{y}-1\right)\;dy+\Gamma \alpha .$$

The first term in (18) describes the contribution from crosslinks that were present at *t* = 0 and the second from those that formed on *y* = 1 in *t* > 0; the third term represents the pectin matrix. At large times, the crosslinks initially present are subject to large stretching (promoting breakage) and are ultimately confined to an exponentially narrowing region near the outer surface of the cell wall (region A, figure 3); while this group of crosslinks plays an important role in the initial build-up of stress in the wall, it has a negligible contribution at large times (because highly elongated crosslinks break almost surely via (8)), and the stress resultant approaches its steady-state limit

(19) $$\Sigma_{\infty }=\int_{0}^{1}\exp\left(-\frac{G(y)}{\alpha }\right)\left(\frac{1}{y}-1\right)\;dy+\Gamma \alpha .$$

**Fig. 4:**
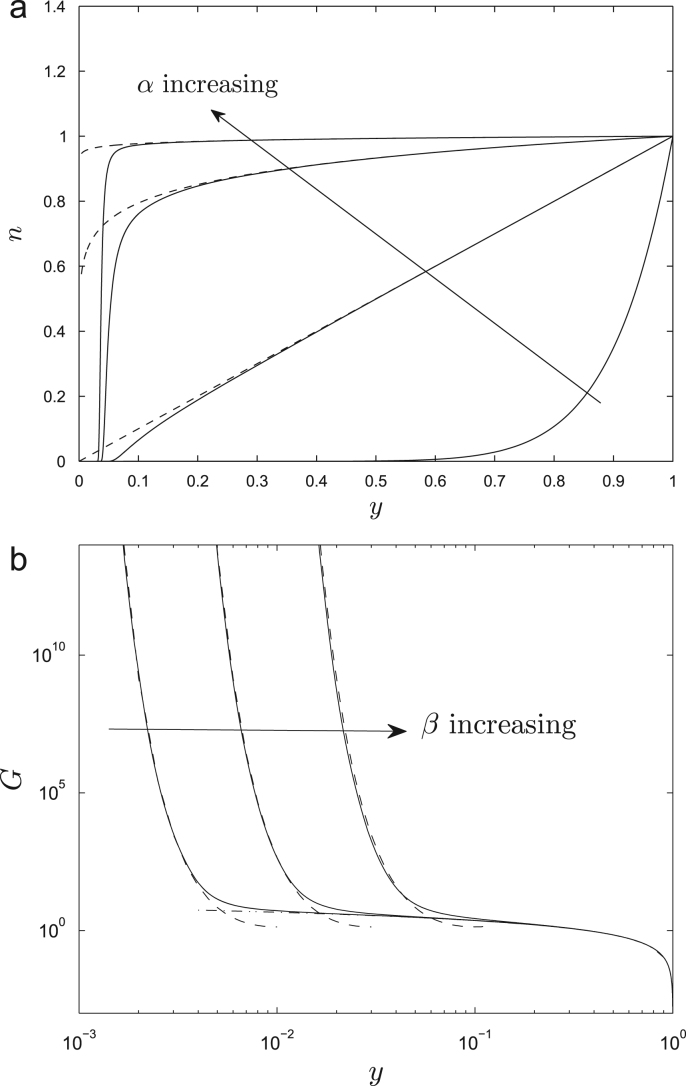
(a) Crosslink density *n*(*y*) (solid) for *α* = 0.1, 1, 10, 100 and *β* = 0.1, with no enzyme, satisfying (17) in 0 < *y* ≤ 1. Dashed lines show the approximation *n* ≈ *y*^1/*α*^. For *α* ≥ 1, *n*(*y*) falls rapidly to zero within the scission layer near *y* = 0. (b) *G*(*y*) (solid) compared to asymptotic limits (20) (dashed, dot-dashed) for *β* = 0.01, 0.03, 0.1.

Figure 4(a) illustrates typical steady-state crosslink distributions satisfying (17). These can be understood by examining the limit *β* ≪ 1, when (16) may be approximated by

(20) $$G(y)\approx \left\{\begin{array}{cc} \log(1/y) & \beta \ll y\leq 1,\\ (y^{2}/2\beta^{2})e^{\beta^{2}/y^{2}} & 0<y\ll \beta , \end{array}\right)$$

as illustrated in Figure 4(b). It follows that *n*(*y*) is exponentially small for 0 < *y* ≪ *β* and that *n* ≈ *y*^1/*α*^ outside this ‘scission layer,’ as illustrated in Figure 4(a). The threshold strain rate *α*_*T*_ = 1 therefore distinguishes two types of behaviour (Figure 4a): under rapid stretching (*α* ≫ *α*_*T*_, e.g. *α* = 100 in Figure 4a), crosslinks remain intact (*n* ≈ 1) outside the scission layer; under weak stretching (*α* ≪ *α*_*T*_, e.g. *α* = 0.1 in Figure 4b), crosslinks are localised near the inner surface of the wall at *y* = 1, breaking before they are carried towards the outer surface of the wall by the stretching flow. The stress resultant (18) will therefore reach its large-time limit (19) when the crosslinks that are initially present in the wall (region A in Figure 3) are confined within the scission layer, i.e. for *t* ≫ (1/*α*) log(1/*β*), i.e. the steady state equilibrates quicker under large strain rates. This behaviour is illustrated via simulations in Section 3 below.

### 2.3 Incorporating XTH enzyme action

To describe XET and XEH actions we now restrict attention to steady-state crosslink distributions, writing *n* = *n*(*y*). Since the wall is thin, we assume for simplicity that enzymes are able to diffuse sufficiently fast that their action can be taken to be spatially homogeneous through the wall.

We incorporate additional enzyme-mediated breakage and reformation by replacing (7) (expressed in dimensional variables) with

(21a) $$-\alpha^{*}y^{*}\frac{dn^{*}}{dy^{*}}=-k_{\mathrm{off}}^{*}n^{*}-k_{0}(g_{\mathrm{XEH}}+g_{\mathrm{XET}})n^{*},\;(0\leq y^{*}<h)$$

subject to

(21b) $$n^{*}(h)=n_{0}+\frac{k_{0}g_{\mathrm{XET}}n^{\mathrm{tot}}}{\alpha^{*}h},\;n^{\mathrm{tot}}\equiv \int_{0}^{h}n^{*}(y^{*})\;dy^{*}.$$

The non-negative factors *g*_XET_, *g*_XEH_ represent respectively the action of XET and XEH in enhancing bond breakage. (Enzyme-mediated bond breakage is here described with linear additive terms; we find qualitatively similar behaviour using instead terms of the form $k_{\mathrm{off}}^{*}(g_{\mathrm{XEH}}+g_{\mathrm{XET}})$.) We assume that every crosslink that is broken under XET enzyme action is immediately reformed at its unstressed length and that reformed crosslinks are deposited at the inner surface of the wall. The precise form of the boundary condition (2.3b) is justified in A.

We nondimensionalise (2.3) as in (11), recovering

(22a) $$-\alpha y\frac{dn}{dy}=-\exp\left(\beta^{2}{\left(y^{-1}-1\right)}^{2}\right)n-(g_{\mathrm{XEH}}+g_{\mathrm{XET}})n,$$

(22b) $$n(1)=1+\frac{g_{\mathrm{XET}}}{\alpha }\int_{0}^{1}n\;dy.$$

It follows that

(23a) $$n=\frac{y^{(g_{\mathrm{XEH}}+g_{\mathrm{XET}})/\alpha }}{1-A}\exp\left(-\frac{G\left(y\right)}{\alpha }\right),$$

(assuming 0 ≤ *A* < 1) where *G*(*y*) is given in (16) and

(23b) $$A=\frac{g_{\mathrm{XET}}}{\alpha }\int_{0}^{1}\exp\left(-\frac{G(y)}{\alpha }\right)y^{\left(g_{\mathrm{XEH}}+g_{\mathrm{XET}}\right)/\alpha }\;dy.$$

Equation (2.3) reduces to (17) when *g*_XET_ = *g*_XEH_ = 0. Using (2.3), the steady-state stress resultant becomes

(24) $$\Sigma_{\infty }=\frac{1}{1-A}\int_{0}^{1}\exp\left(-\frac{G(y)}{\alpha }\right)y^{\left(g_{\mathrm{XEH}}+g_{\mathrm{XET}}\right)/\alpha }\left(\frac{1}{y}-1\right)\;dy+\Gamma \alpha .$$

Numerical predictions of Σ_∞_ versus *α* are given in Section 3 below.

It is again instructive to seek an approximation of (2.3) when *β* ≪ 1. Motivated by (20) and figure 4, we adopt the approximation

(25) $$\exp\left(-\frac{G(y)}{\alpha }\right)\approx \left\{\begin{array}{cc} y^{1/\alpha } & C\beta \leq y\leq 1,\\ 0 & 0<y<C\beta , \end{array}\right)$$

for some constant *C* = *O*(1). It follows from (2.3b) that

(26) $$A\approx \frac{g_{\mathrm{XET}}}{\alpha +\alpha_{0}}\left(1-{\left(C\beta \right)}^{(\alpha_{0}+\alpha )/\alpha }\right),\;\alpha_{0}\equiv 1+g_{\mathrm{XET}}+g_{\mathrm{XEH}},$$

and hence from (2.3a) that

(27) $$n\approx \frac{(\alpha +\alpha_{0})y^{\alpha_{0}/\alpha }H\left(y-C\beta \right)}{\alpha +\alpha_{0}-g_{\mathrm{XET}}\left(1-{\left(C\beta \right)}^{(\alpha_{0}+\alpha )/\alpha }\right)},$$

where *H* is the Heaviside function. Eq. (27) suggests that enzyme-mediated crosslink breakage increases the threshold strain rate from *α*_*T*_ = 1 (as in Figure 4(a)) to *α*_*T*_ = *α*_0_, such that *n* decays close to the inner surface of the wall *y* = 1 for *α* ≪ *α*_*T*_ while *n* remains almost uniform across most of the cell wall for *α* ≫ *α*_*T*_. Using (25) and (27), the steady-state stress resultant (24) can be approximated by

(28) $$\begin{array}{cc} \Sigma_{\infty }\approx & \frac{\alpha }{\alpha +\alpha_{0}-g_{\mathrm{XET}}\left(1-{\left(C\beta \right)}^{(\alpha_{0}+\alpha )/\alpha }\right)}\left[\frac{\alpha +\alpha_{0}}{\alpha_{0}}\left(1-{\left(C\beta \right)}^{\alpha_{0}/\alpha }\right)\right)\\ & \;\left(+\left({\left(C\beta \right)}^{\left(\alpha +\alpha_{0}\right)/\alpha }-1\right)\right]+\Gamma \alpha . \end{array}$$

Writing L≡log(1/Cβ), (28) can be re-expressed (in the absence of enzyme) as

(29) $$\Sigma_{\infty }\approx \alpha \left(1-e^{-L/\alpha }\right)+\frac{\alpha }{\alpha +1}\left(e^{-L(\alpha +1)/\alpha }-1\right)+\Gamma \alpha .$$

We test the accuracy of (28) and (29) below, with and without enzyme effects respectively. An alternative modification of the model to simulate the effect of expansins is given in B.

## 3 Results

### 3.1 Steady elongation

**Fig. 5:**
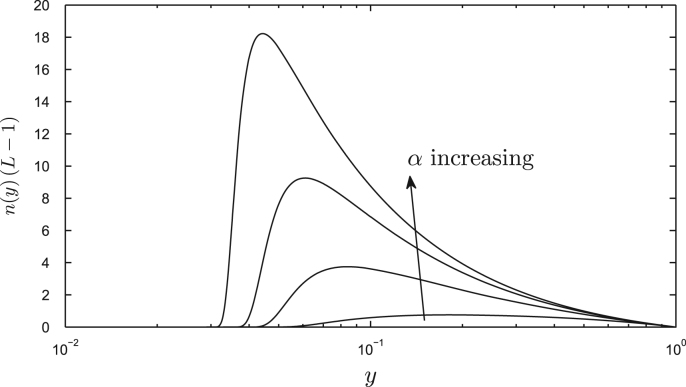
The distribution of stress due to cross-links across the cell wall (note logarithmic scale for *y*) for *β* = 0.1, for *α* = 1, 3, 10, 100, with no enzyme.

We first consider the steady-state behaviour of the enzyme-free model (Section 2.2). Figure 5 illustrates the distribution of stress within the wall provided by stretched crosslinks. This is given by *n*(*L* − 1), i.e. the integrand in (19); recall that the distribution of *n* across the wall for *β* ≪ 1 is shown in Figure 4(a). The maximum stress arises deep within the wall, between the scission layer near the outer surface (where all crosslinks have broken) and the inner surface of the wall (where crosslinks are laid down in their unstressed configuration). For *α* = 1, for example, the rate of crosslink stretching balances the rate of breakage, allowing crosslinks to share the stress uniformly across much of the wall; for *α* > 1, however, crosslinks are stretched quickly, remaining intact across much of the wall, breaking only in the scission layer, the stress being dominated by a small number of highly stretched crosslinks that are just about to break.

**Fig. 6:**
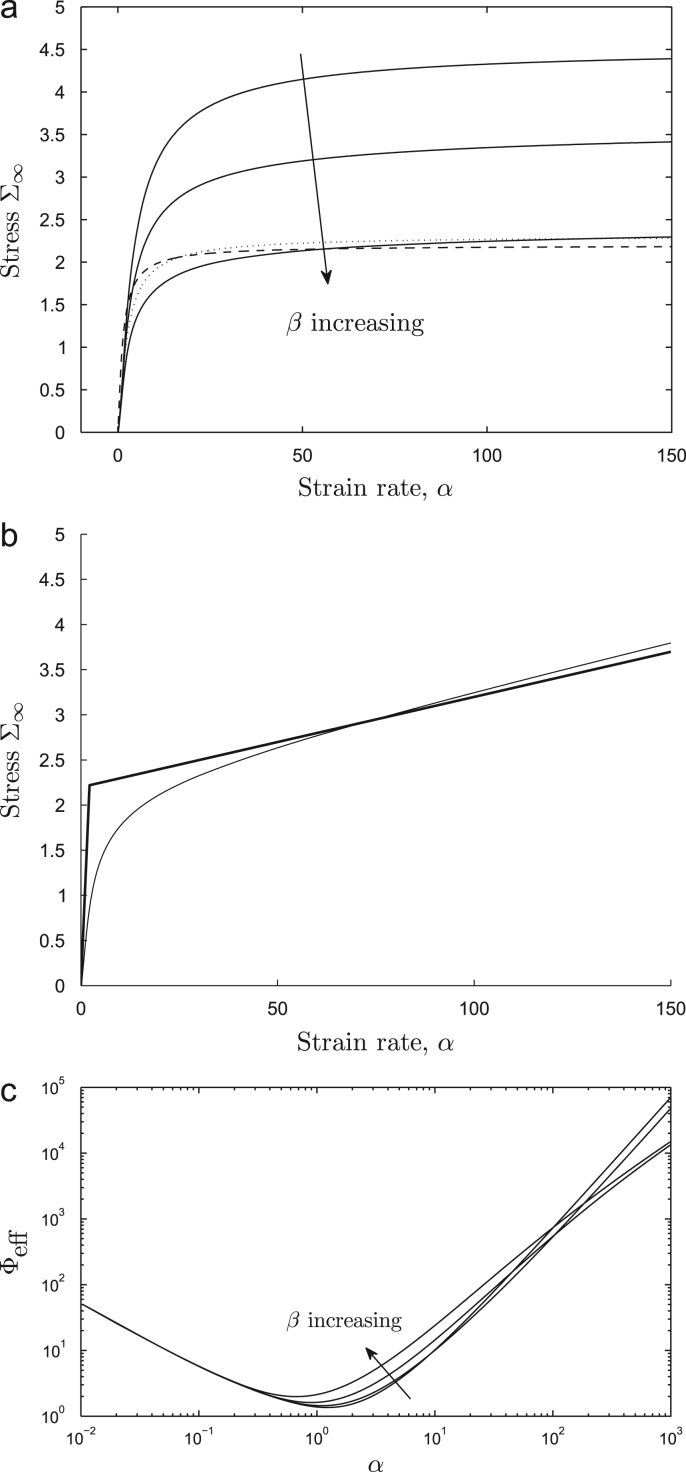
(a,b) Steady-state stress resultant versus strain-rate, given by (19) with (a) Γ = 0, *β* = 0.01, 0.03, 0.1 (solid) and asymptotic approximations (29) (dotted, with *Cβ* = 0.03786) and (30) (dashed, with L=2.198) and (b) including matrix viscosity Γ = 0.01, *β* = 0.1; the heavy line in (b) shows Lockhart-type behaviour exhibited by (31). (c) Effective extensibility Φ_eff_ versus strain rate *α* for *β* = 0.001, 0.01, 0.03, 0.1 and Γ = 0.

Integrating this stress distribution across the wall gives the steady-state stress resultant (19), which we plot for Γ = 0 in Figure 6(a) and with Γ = 0.01 in Figure 6(b). Two regimes are evident, that we may identify as pre-yield and post-yield states. At small strain rates (e.g. *α* < 5), the stress resultant rises steeply with *α*, indicating very low effective extensibility $\Phi_{\mathrm{eff}}^{*}$ (see (3)) corresponding to the pre-yield state. For this range of *α*, as indicated by Figure 4(a), a small increase in strain rate allows more crosslinks to remain intact within the wall before breakage occurs; the additional crosslinks elongate as they penetrate the wall, supporting an increased stress resultant that is distributed across many crosslinks. For *α* above unity, there is significant stretching of crosslinks, enabling a greater stress to be borne (Figure 5). At larger strain rates (e.g. *α* > 10), almost all crosslinks remain intact until there is abrupt rupture very close to the outer surface of the wall (Figure 4a); in this case a small increase in *α* leads to only a small increase in the number of intact crosslinks (the system being close to saturation) and hence to only a small increase in stress. The contribution to Σ_∞_ due to crosslinks therefore reaches a plateau for large *α* (Figure 6a) and the effective extensibility due to crosslinks in the post-yield state is therefore high, although the magnitude of Σ_∞_ is greater than that for smaller *α*. Increasing *β* (i.e. reducing the length to which crosslinks can extend before they break) leads to a modest reduction in the post-yield stress resultant but retains the strong saturation effect (Figure 6a). When Γ > 0 (Figure 6b), it is evident that the post-yield extensibility is dominated by the pectin matrix at very large strain rates.

Figure 6(c) shows how the effective extensibility (3) varies with *α* for Γ = 0. Here we plot its dimensionless analogue $\Phi_{\mathrm{eff}}=(E/k_{0})\Phi_{\mathrm{eff}}^{*}$. Again it is evident that the wall is very stiff when *α* is order unity, but Φ_eff_ increases by three orders of magnitude (and the wall softens substantially) as *α* increases to 100; the extensibility is only weakly sensitive to *β* when *β* ≪ 1. The model also predicts that the wall is soft for *α* ≪ 1, but in this range the stress is vanishingly small and is not regarded as physiological.

Because the parameter dependence in (16, 19) is not obvious, it is helpful to consider the approximation (29) for Σ_∞_ in the limit *β* → 0. Figure 6(a) shows that (29) captures well the behaviour of (19) for *β* = 0.1 (for a suitable choice of *C*). While in practice *β* may not be sufficiently small for L to become very large, further insight arises by considering the limiting form of (29) for large L. Motivated by the argument of the first exponential in (29), we set α=Lξ, taking L≫1, *ξ* = *O*(1). Then (29) simplifies at leading order to

(30) $$\Sigma_{\infty }\approx L\xi \left(1-e^{-1/\xi }+\Gamma \right)=\alpha \left(1-e^{-L/\alpha }\right)+\Gamma \alpha .$$

This is plotted in Figure 6(a) for *β* = 0.1, Γ = 0, again showing good agreement with (19) while being considerably easier to interpret. In particular, (30) shows how

(31) $$\Sigma_{\infty }\approx \left\{\begin{array}{cc} (1+\Gamma )\alpha & 1\ll \alpha \ll L\\ L+\Gamma \alpha & \alpha \gg L, \end{array}\right)$$

which is plotted using heavy lines in Figure 6(b). This piecewise linear approximation captures well the behaviour of Σ_∞_ for large and small values of *α*, and recapitulates the Lockhart equation (1), bar the difference between zero and small extensibility in the pre-yielded state.

Equation (31) shows that, in the pre-yield state, Σ_∞_ rises steeply at a rate approximately independent of *β*, consistent with in Figure 6(a). Assuming Γ ≪ 1, the corresponding (dimensional) effective extensibility due to cross-links $\Phi_{\mathrm{eff}}^{*}$ (see (3)) can therefore be approximated as

(32) $$\Phi_{\mathrm{eff}}^{*}\approx \frac{k_{0}}{n_{0}\kappa L_{0}h}\equiv \frac{k_{0}}{E}.$$

(This corresponds to the minimum Φ_eff_ ≈ 1 in Figure 6(c).) Thus low pre-yield wall extensibility is ensured by slow bond breakage (low *k*_0_) and a high density of stiff crosslinks (large *n*_0_ and *κ*_0_). In contrast, for large *α*, (31) shows that the post-yield crosslink contribution to the stress resultant saturates at a level that depends weakly (only logarithmically) on *β* (again evident in Figure 6a,c). The transition between these two types of behaviour takes place for strain rates *α* of magnitude L (30, 31), i.e. at a value of *α* that is only weakly sensitive to *β*. We can also use (31) to estimate the magnitude of the yield stress in (1): in dimensional variables, in the post-yield state we can re-express (1) as $\Sigma^{*}=Y+\alpha^{*}/\Phi_{\mathrm{eff}}^{*}$, from which, by comparison with (31), we infer

(33) $$Y\approx EL,\;\Phi_{\mathrm{eff}}^{*}\approx \Phi_{m}.$$

This confirms that the kinetics of crosslink breakage (encompassed in L) are central to the origins of yield stress (although *Y* depends only weakly on the crosslink extension parameter *β*), and that the matrix dominates the post-yield extensibility. Consistent with the Veytsman–Cosgrove model (Veytsman and Cosgrove, 1998), the yield stress increases with increased density and stiffness of crosslinks.

In summary, the transition from a stiff structure at small *α* (with small pre-yield $\Phi_{\mathrm{eff}}^{*}$, see (32)) to a relatively soft structure at large *α* is characteristic of a Bingham material, and suggests how crosslink stretching and breaking can infer the stretching cell wall with an effective yield stress. However, as Figure 6 illustrates, the predicted yield transition is smoother than suggested by (1) or (31). Nevertheless, if one adopts the simple Lockhart description, then to a first approximation for *β* ≪ 1 our model predicts that the matrix viscosity determines low post-yield effective extensibilty and the crosslinks determine the yield stress in (33). We emphasise however that in practice, given the limitations of the approximation (30) for realistic parameter values, there are likely to be contributions of both crosslinks and matrix to *Y* and Φ.

### 3.2 Unsteady effects

**Fig. 7:**
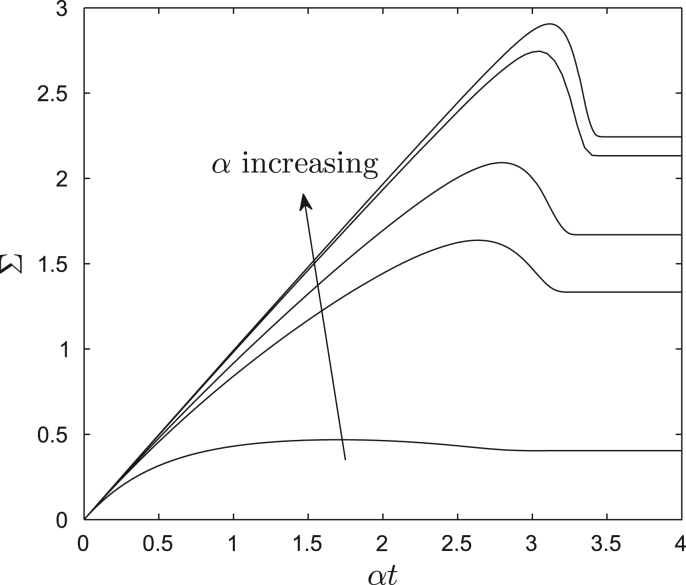
Time-evolution of stress under fixed strain-rate; *αt* is log of the wall stretch and the wall is initially unstressed, *β* = 0.1, Γ = 0, *L*_1_ = 1, *α* = 1, 5, 10, 50, 100.

The steady-state relationship between Σ and *α* in Figure 6 reflects predominantly viscous behaviour: the motion is dissipative and irreversible, relying on a balance between crosslink formation and breakage. However at early times, before there is substantial bond breakage, the wall exhibits additional elastic behaviour, as crosslinks present at *t* = 0 are stretched. This stress relaxation is captured in (18) and plotted against *αt* (log of the wall stretch) for constant strain rate *α* and Γ = 0 in Figure 7. In this simple example the wall is initially unstressed. By extending an initially uniform distribution of crosslinks, the stress resultant rises and in general overshoots its large-time limit.

For small stretch (*αt* ≪ log(1/*β*)), we can use (20) in (18) to show that

(34) $$\Sigma \approx \alpha t-{\left(\alpha t\right)}^{2}/\alpha .$$

Since the Cauchy strain of the elongating wall is given by $e^{\alpha t}-1\approx \alpha t+\frac{1}{2}{\left(\alpha t\right)}^{2}$, (34) demonstrates intially linearly elastic behaviour followed by strain softening. Expressing (34) in dimensional terms, we find that the extensional stiffness of the wall at small strain is (as anticipated) $E\equiv n_{0}\kappa L_{0}h$. The transient maximum stress resultant provides an alternative measure of the cell wall's yield stress; the corresponding value of *αt* can be used to deduce the so-called yield strain (Groot et al., 1996; Moller et al., 2006). It is evident from Figure 7 that the yield strain increases with strain rate for small *α* but saturates for large *α* (when *αt* ≈ 3.2 in this case). Yielding can be identified with breakage of the bonds that were initially present as they enter the scission layer at the outer wall (Section 2.2), which occurs when *αt* = *O*(log(1/*β*)) (Figure 3), consistent (when *α* is large) with the estimate Y≈EL in (33). The crosslinks can therefore be considered as acting as a Maxwell viscoelastic element (elastic and viscous units in series), albeit with nonlinear and strongly coupled components.

### 3.3 XTH enzyme action

**Fig. 8:**
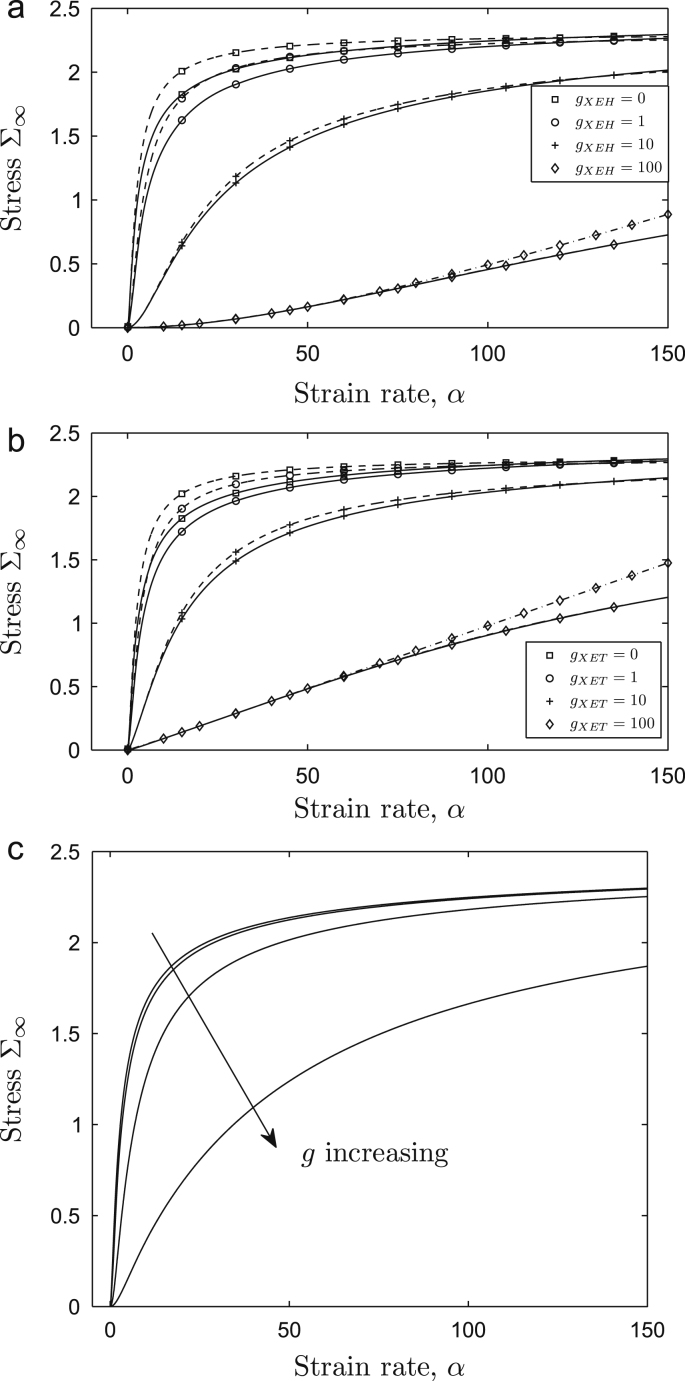
The effect of enzyme on steady-state stress resultant versus strain rate under (a) XEH, (b) XET and (c) expansin action. (a, b) show (24) (solid), (28) (dashed) for *β* = 0.1 and Γ = 0 with (a) *g*_XEH_ = 0, 1, 10, 100 and *g*_XET_ = 0, (b) *g*_XET_ = 0, 1, 10, 100 and *g*_XEH_ = 0; the dot-dashed curves with diamonds shows (38). (c) shows (39), computed using (48), for *β* = 0.1, Γ = 0, using *g* = 0, 1, 10, 100.

We now consider how the presence of enzymes of the XTH family affects the steady-state crosslink dynamics, using results of Section 2.3. Figures 8(a,b) show that both XEH and XET action decrease the stress resultant (24) for a given strain rate, with a larger effect observed for XEH action since XET action increases both breakage rate and crosslink formation. The sharp transition between pre-yield and post-yield states becomes less evident as the enzyme activity increases because of wall softening in the pre-yield state. However Σ_∞_ (with Γ = 0) again saturates for very large *α*. To interpret Figures 8(a,b) in their biological context, one can imagine a segment of cell wall subject to fixed axial stress Σ_∞_ ≈ 1, say, imposed by cell turgor. In the presence of XET enzyme, the strain rate increases from *α* ≈ 4 (*g*_XET_ = 0) to *α* ≈ 115 (*g*_XET_ = 100), enabling rapid cell elongation.

Figures 8(a,b) also demonstrate the accuracy of the approximation (28) when *β* ≪ 1. Further simplification, assuming L≫1 and *α*_0_ = *O*(1), yields the leading-order relation

(35) $$\Sigma_{\infty }\approx \frac{\alpha }{\alpha_{0}}\left(1-e^{-L\alpha_{0}/\alpha }\right)+\Gamma \alpha$$

when α=O(L). At this level of approximation, the distinction between XET and XEH action is not evident (bond breakage being the dominant effect). Equation (35) implies

(36) $$\Sigma_{\infty }\approx \left\{\begin{array}{cc} (\alpha_{0}^{-1}+\Gamma )\alpha & 1\ll \alpha \ll \alpha_{0}L\\ \Sigma_{\infty }\approx L+\Gamma \alpha & \alpha \gg \alpha_{0}L. \end{array}\right)$$

Thus, in this approximation, XTH enzymes do not alter the maximum stress carried by crosslinks for very large strain rates. However in the pre-yield range $1\ll \alpha \ll \alpha_{0}L$, the (dimensional) effective extensibility (3) satisfies

(37) $$\frac{1}{\Phi_{\mathrm{eff}}^{*}}\approx \frac{E}{k_{0}(1+g_{\mathrm{XET}}+g_{\mathrm{XEH}})}+\frac{1}{\Phi_{m}}.$$

This shows how, under fixed stress Σ_∞_, the wall can soften in the presence of XET and XEH action, increasing the small effective extensibility in the pre-yield state (32) roughly proportionally to *g*_XET_ + *g*_XEH_ so that the expansion rate of the cell increases accordingly.

When enzyme action is strong (*α*_0_ ≫ 1), the distinction between XET and XEH action becomes more evident (Figures 8a,b). We can demonstrate this via an approximation of (28) valid for $\alpha \ll L\alpha_{0}$, namely

(38) $$\Sigma_{\infty }\approx \frac{\alpha^{2}}{\alpha_{0}(\alpha +1+g_{\mathrm{XEH}})}+\Gamma \alpha ,$$

which is plotted for (*g*_XEH_, *g*_XET_) = (100, 0) and (0, 100) and Γ = 0 in Figures 8(a) and (b) respectively. The stress resultant exhibits a transition between quadratic and linear *α*-dependence for *α* = *O*(1 + *g*_XEH_), as is evident in Figure 8(a). While the extensibility for large *α* in (38) matches that captured in (37) (so that both large-*α*_0_ curves have slope 1/*α*_0_ for large *α* in Figure 8), bond reformation allows the wall to sustain larger stress in the XET case.

### 3.4 Expansin action

Finally, Figure 8(c) shows the predicted stress/strain-rate relationship in the presence of enzymes of the expansin family. Here the model has been modified to simulate unpeeling of crosslinks from CMF, allowing stress relaxation in crosslinks by increase of the crosslinks’ rest length *L*_0_; this results in the stress/strain-rate relation (19) being modified as

(39) $$\Sigma_{\infty }=\int_{0}^{1}\exp\left(-\frac{G(y)}{\alpha }\right)\left(\frac{1}{y}-f_{0}\left(\frac{1}{y}\right)\right)\;dy+\Gamma \alpha ,$$

where *f*_0_(*L*) (1 ≤ *f*_0_ ≤ *L*) represents the increase in the unstressed length *L*_0_ of a crosslink as it elongates. A candidate form of *f*_0_ is provided in B, with details of the derivation of (39); *G* in (39) takes the modified form given in (50). The model predicts broadly similar qualitative behaviour to that exhibited by XTH enzymes, with the primary effect of enzyme being a reduction in the pre-yield extensibility.

## 4 Discussion

The complex and dynamic structure of the plant cell wall presents significant challenges in constitutive modelling. Here we have taken a deliberately simple approach in order to understand how hemicellulose crosslinks contribute to the mechanical properties of the wall, particularly the regulation provided by enzymes exhibiting XET and XEH activity. In our model we have assumed that CMF are oriented perpendicular to the direction in which the wall is elongating, so neglecting shearing and torsion due to helical fibre orientations, and that crosslinks are oriented orthogonal to the CMF. We have represented the crosslinks as linear springs, whereas they are likely to be nonlinear, for example exhibiting strain-stiffening, and may also interact with the pectin matrix as they unfold (Abasolo et al., 2009). We have also assumed that the CMF, wall matrix and crosslinks are laid down on the inner wall of the cell so as to ensure that the wall thickness remains constant and the density of each component remains uniform across the wall; the model can readily be extended to include a more detailed description of deposition processes, and could reveal how such metabolic factors might influence cell and tissue expansion rates. We have also adopted an elementary model of enzyme-mediated crosslink kinetics, including a simplifying assumption that crosslink formation is confined to the inner surface of the cell wall. While these assumptions are all open to debate in particular circumstances, and merit reassessment in future studies, it is nevertheless useful to explore their consequences. Furthermore, the relatively simple model has enabled us to derive rationally-based analytic expressions for wall properties that reveal underlying biophysical mechanisms. By developing a generic model, we provide a framework that could be adapted to incorporate features appropriate to specific plant organs.

Our model incorporates the extensional flow of the pectin matrix within the elongating wall, and the associated transport of CMF and crosslinks with the matrix, such that individual crosslinks elongate as they are driven towards the outer surface of the cell wall. As a consequence, when the wall is elongating rapidly the extensional stress is distributed nonuniformly through the wall, with a maximum close to the outer wall (Figure 5). This is consistent with observations (Hejnowicz and Borowska-Wykret, 2005) showing how relaxation of tensile stress (via detachment) induces buckling of the inner wall of sunflower hypocotyl, indicating that the outer wall transmits the majority of the tensile stress in the wall.

Combining our description of crosslink transport through the wall with a representative model for force-enhanced crosslink breakage (8), we have derived a relationship between stress and strain rate in the wall that is strongly nonlinear, recovering the distinctive yielding behaviour of the Lockhart model (1), as illustrated in Figure 6(b). The main origin of nonlinearity is the assumption that crosslinks stretch by up to a factor 1/*β*, where *β* ≪ 1, before breaking rapidly (see (8)). The Lockhart equation (1) has the advantages of simplicity (being piecewise linear) and parsimony (being described by just two phenomenological parameters, extensibility Φ and yield *Y*). The present model suggests that the relationship between stress and strain rate in the cell wall may not vary as sharply between pre- and post-yield states as suggested by the Lockhart equation (Figure 6b), making it harder to identify a well-defined yield stress. However to a first approximation, our model suggests that: (i) the low pre-yield extensibilty is determined by a balance between extension and slow breakage of crosslinks that have not undergone substantial stretch (32); (ii) yield arises from elastic stresses in crosslinks that have reached their maximum extension (33)_1_; (iii) the wall's high post-yield extensibilty is determined primarily by the matrix (33)_2_.

In addition to demonstrating viscoplastic behaviour characteristic of a Bingham material over long timescales, our model also demonstrates how crosslinks contribute to viscoelastic behaviour of the cell wall over short timescales. For an element of cell wall initially at rest but then subject to a fixed strain rate, our model shows (Figure 7) how the stress exhibits a transient overshoot (as crosslinks are stretched rapidly) followed by stress relaxation (as extended crosslinks break). Again assuming *β* ≪ 1, we demonstrate how the wall's (elastic) extensional stiffness E contributes in a simple way to the pre-yield extensibility $\Phi_{\mathrm{eff}}^{*}$ (32) and the yield stress *Y* (33)_1_. (Interestingly, a similar relationship connecting effective viscosity to Youngs modulus and rate of crosslink dissociation was reported in (Rojas et al., 2011) in a model of the pectin wall of a pollen tube.) Indeed it might be possible to use the prediction $Y/E\approx \log(L_{\max}/{\mathrm{CL}}_{0})$ to infer molecular properties of crosslinks from macroscopic measurements. Our model could be adapted to demonstrate transient creep under a fixed load but this involves a more complex calculation that we have not pursued here.

Given the many approximations inherent in the model, and the difficulty of measuring key parameters, it is fruitful to test the model at a qualitative than quantitative level. For example, Takeda et al. (2002) showed how incorporation of whole xyloglucan molecules into the cell wall of pea stem segments suppressed cell elongation, whereas incorporation of fragments accelerated it. In the former case, we can interpret incorporation of xyloglucan as increasing the number density of crosslinks *n*_0_, which increases E and hence the pre-yield extensibility $\Phi_{\mathrm{eff}}^{*}$ and yield stress, leading to growth suppression. In the latter case, incorporation of fragments into existing cross-links can be interpreted as increasing *L*_0_, which provides a mechanism of stress relaxation and hence accelerated growth. There is significant scope for extending the model to incorporate the potential regulatory role of the deposition processes, which would introduce metabolic factors to the model that are potentially under experimental control.

We have also used our model to investigate the effect of enzyme action on the cell wall. Both XET and XEH modes of enzyme action reduce the effective pre-yield extensibility of the wall, as illustrated by Figure 8(a,b) and captured approximately by (37). This behaviour enables the cell wall to elongate much more rapidly for a given stress. The model suggests that the two modes of enzyme action can exhibit broadly similar behaviour, suggesting that, at low levels of expression, it may be difficult to identify phenotypic differences between XET and XEH actions. Differences between the two modes of enzyme action emerge in model predictions only when crosslink-breakage rates are elevated substantially above baseline values by enzyme, in which case XEH produces larger growth rates for the same imposed stress than XET ((38), Figure 8). We emphasise that these predictions are dependent on numerous assumptions and are at best indicative of likely behaviour, and substantial further work is required to test their quantitative accuracy. However qualitative support comes from experiments in which the locations of XTH enzymes have been correlated with regions of rapid tissue expansion (Vissenberg et al., 2000), and studies showing increased elongation rate after addition of exogenous XTH (Van Sandt et al., 2007). Within a growing organ, these different modes of enzyme action may have complementary roles or be upregulated within different growth regions in order to provide tight control of growth rates.

In our model we have considered crosslinks to be either bound or unbound, whereas crosslinks may progressively unpeel from CMF as the CMF move apart (Jarvis, 2009), providing an alternative mechanism for stress relaxation in the wall. By promoting unpeeling, expansins are believed to soften the wall, hence effectively increasing the unstressed length of each crosslink. As illustrated in Figure 8(c), our model predicts that expansin action broadly mirrors XTH action, by increasing the pre-yield extensibility. It would also be possible to consider the action of PME on the matrix extensibility Γ and matrix yield, although we have not pursued this here.

At a conceptual level, our overall approach follows ideas proposed by Passioura and Fry (1992), but our implementation is more systematic. By tracking the evolution of crosslinks as they move through the elongating wall, we can resolve intramural stress inhomogeneities. We also provide detailed candidate mechanisms for the origins of the wall's material properties in terms of molecular binding kinetics, rather than an empirical microscopic yield parameter. From a modelling perspective, it is also more systematic to have constitutive equations that are not explicit functions of time (as in Passioura and Fry, 1992), instead allowing time-dependence to emerge naturally by analysing transport along characteristics. An important area that we do not address directly here is the role of PME in controlling stiffness through its action on the pectin matrix (Boyer, 2009), and it will be interesting in future models to assess the role of calcium crosslinking (following for example (Rojas et al., 2011)) alongside the hemicellulose crosslinking considered here. It will also be important to embed models of pectin and hemicellulose crosslinking into constitutive models that account for reorientation of microfibrils (Dyson and Jensen, 2010) to understand fully how the composition of the cell wall determines its mechanical properties.

In conclusion, our microstructural model has shown how yield in the expanding plant cell wall can be explained in terms of the evolving cell wall microstructure. Our model suggests that the nonlinearity inherent in the Lockhart equation can be traced to the mechanochemical kinetics underpinning crosslink breakage. We believe this model will provide a useful bridge in understanding the action of enzymes on growth regulation at the scale of complete plant tissues and organs.

## Appendix A The XET boundary condition

To incorporate the effect of XET and XEH enzymes, (7) (expressed in dimensional variables) is initially replaced with

(40) $$-\alpha^{*}y^{*}\frac{dn^{*}}{dy^{*}}=\left\{\begin{array}{cc} -k_{\mathrm{off}}^{*}n^{*}-k_{0}(g_{\mathrm{XEH}}+g_{\mathrm{XET}})n^{*}, & (0\leq y^{*}<h-\delta h),\\ (k_{0}/\delta h)g_{\mathrm{XET}}\int_{0}^{h}n^{*}\;dy^{*}, & (h-\delta h<y^{*}<h). \end{array}\right)$$

Every crosslink that is broken under XET enzyme action is immediately reformed at its unstressed length; to allow analytical progress, we assume that crosslinks are reformed within a thin layer of width *δh* on the inner surface of the cell wall. Then, integrating (40) between *y*^*^ = *h* − *δh* (where *n*^*^ = *n*_0_ + *N*_0_, say) and *y*^*^ = *h* (where *n*^*^ = *n*_0_), we find

(41) $$-\int_{n_{0}+N_{0}}^{n_{0}}\;dn^{*}=\frac{k_{0}g_{\mathrm{XET}}}{\alpha^{*}\delta h}\int_{0}^{h}n^{*}\;dy^{*}\int_{h-\delta h}^{h}\frac{\;dy^{\prime }}{y^{\prime }}.$$

Taking the limit *δh* → 0, (40) reduces to the modified Smoluchowski equation (2.3).

## Appendix B Simulating the action of expansin

Expansins are believed to target the hydrogen bonds that connect hemicellulose crosslinks to CMF (Burgert and Fratzl, 2007). The ‘molecular velcro’ hypothesis (Jarvis, 2009) suggests that crosslinks may unpeel from CMF, without completely disrupting the bond but leading to stress relaxation in individual crosslinks. In our model of a crosslink as a linear spring, we can simulate unpeeling by increasing the spring's unstressed length in response to stretch. We briefly investigate the consequence of this within the context of our simplified structural model.

We define $L_{0}^{*}$ to be a variable representing the evolving unstressed length of a crosslink. One possible model for expansin action is based on the assumption that the unpeeling rate is proportional to the force exerted by the extended bond, i.e.

(42) $$\frac{dL_{0}^{*}}{dt^{*}}=g_{\mathrm{EXP}}^{*}(L^{*}-L_{0}^{*}),$$

where $g_{\mathrm{EXP}}^{*}$ is a rate constant dependent on expansin concentration. Nondimensionalising as in (11), but now scaling lengths on $\bar{L}$, the length of unstressed bonds deposited on the inner surface of the cell wall, the model equations for steady elongation (2.2) become

(43) $$\frac{dL}{dt}=\alpha L,\;\frac{dL_{0}}{dt}=g(L-L_{0})$$

where $g=g_{\mathrm{EXP}}^{*}/k_{0}^{*}$, with

(44) $$\frac{dn}{dt}=-n\exp\left(\beta^{2}\sigma {\left(L-L_{0}\right)}^{2}\right),$$

on d*y*/d*t* = − *αy*, with

(45) $$\Sigma \left(t\right)=\int_{0}^{1}n\left(y,t\right)\left(L-L_{0}\right)\;dy+\Gamma \alpha .$$

Thus, as in (14),

(46) $$\frac{dn}{dL}=-\frac{n}{\alpha L}\exp\left(\beta^{2}{\left(L-L_{0}\right)}^{2}\right),$$

and

(47) $$\frac{dL_{0}}{dL}=\frac{g}{\alpha L}(L-L_{0})$$

with *L* = 1/*y*. Given that *L*_0_ = 1 when *L* = 1, it follows from (47) that

(48) $$L_{0}=f_{0}(L)\equiv \frac{\mathrm{gL}}{\alpha +g}+\left(1-\frac{g}{g+\alpha }\right)L^{-g/\alpha }.$$

This particular functional form is a consequence of our choice of unpeeling kinetics model (42), and other functions *f*_0_ satisfying 1 ≤ *f*_0_ ≤ *L* would emerge from different assumptions on unpeeling kinetics.

We can then integrate (46), expressed as

(49) $$\frac{dn}{dL}=-\frac{n}{\alpha L}\exp\left(\beta^{2}{\left(L-f_{0}(L)\right)}^{2}\right),$$

by redefining *G* in (16) as

(50) $$G(y)\equiv \int_{y}^{1}\frac{\exp\left(\beta^{2}{\left[\left(1/z)-f_{0}(1/z\right)\right]}^{2}\right)}{z}\;dz,$$

to give (17) as before, with (19) becoming (39). Graphs of Σ_∞_, taking *f*_0_ as defined in (48), are plotted versus *α* in Figure 8(c) for increasing values of *g*.
